# Beyond steps per day: other measures of real-world walking after stroke related to cardiovascular risk

**DOI:** 10.1186/s12984-022-01091-7

**Published:** 2022-10-14

**Authors:** Allison Miller, Zachary Collier, Darcy S. Reisman

**Affiliations:** 1 Department of Biomechanics and Movement Science Program, University of Delaware, 540 South College Avenue, Newark, DE 19713 USA; 2 Department of Education and Human Development, University of Delaware, Newark, DE USA; 3 Department of Physical Therapy, University of Delaware, Newark, DE USA

**Keywords:** Stroke, Physical activity, Walking activity, Blood pressure, Real-world monitoring

## Abstract

**Background:**

Significant variability exists in how real-world walking has been measured in prior studies in individuals with stroke and it is unknown which measures are most important for cardiovascular risk. It is also unknown whether real-world monitoring is more informative than laboratory-based measures of walking capacity in the context of cardiovascular risk. The purpose of this study was to determine a subset of real-world walking activity measures most strongly associated with systolic blood pressure (SBP), a measure of cardiovascular risk, in people with stroke and if these measures are associated with SBP after accounting for laboratory-based measures of walking capacity.

**Methods:**

This was a cross-sectional analysis of 276 individuals with chronic (≥ 6 months) stroke. Participants wore an activity monitor for ≥ 3 days. Measures of activity volume, activity frequency, activity intensity, and sedentary behavior were calculated. Best subset selection and lasso regression were used to determine which activity measures were most strongly associated with systolic blood pressure. Sequential linear regression was used to determine if these activity measures were associated with systolic blood pressure after accounting for walking capacity (6-Minute Walk Test).

**Results:**

Average bout cadence (i.e., the average steps/minute across all bouts of walking) and the number of long (≥ 30 min) sedentary bouts were most strongly associated with systolic blood pressure. After accounting for covariates (ΔR^2^ = 0.089, p < 0.001) and walking capacity (ΔR^2^ = 0.002, p = 0.48), these activity measures were significantly associated with systolic blood pressure (ΔR^2^ = 0.027, p = 0.02). Higher systolic blood pressure was associated with older age (β = 0.219, p < 0.001), male gender (β = − 0.121, p = 0.046), black race (β = 0.165, p = 0.008), and a slower average bout cadence (β = − 0.159, p = 0.022).

**Conclusions:**

Measures of activity intensity and sedentary behavior may be superior to commonly used measures, such as steps/day, when the outcome of interest is cardiovascular risk. The relationship between walking activity and cardiovascular risk cannot be inferred through laboratory-based assessments of walking capacity.

## Introduction

Low physical activity is an important modifiable risk factor for stroke and future cardiovascular events [1–5]. In fact, low physical activity may be the second most important modifiable risk factor for stroke, aside from blood pressure [6]. Thus, controlling a person’s risk factors for stroke often involves modifications to their activity behavior to reduce these risks [2]. This is a particularly salient concept in people who have already sustained a stroke who typically demonstrate lower activity levels and greater sedentary behavior compared to persons without stroke [7–11].

The application of sensor technology has enabled rehabilitation professionals to measure real-world activity (i.e., activity that occurs outside the clinic or laboratory setting) to better understand the activity levels of individuals with stroke and its effects on cardiovascular risk [1, 12–14]. In general, two lines of work have garnered significant attention in the sensor field, one related to the measurement of activity and the other focused on understanding predictors of activity. In terms of measurement, the most common way that real-world walking activity has been quantified in stroke rehabilitation studies is by calculating average steps/day (ASPD) [15–19]. Using this measure, a person’s daily stepping activity is monitored over a period of time (preferably at least 7 days) [20, 21], summed and averaged across the number of valid recording days [22, 23]. Thus, ASPD is easy to calculate and interpret, likely contributing to its ubiquitous use in studies in individuals with stroke. There is, however, significant variability in how real-world activity is measured in studies in people with stroke [7, 10, 11, 14, 24–29], with some studies emphasizing the importance of measuring sedentary behavior [11, 25, 28, 30, 31] and others examining measures of activity intensity using metabolic equivalents of task (METS) or cadence [14, 26, 27, 32], among other measures. Thus, it remains unknown *which* measures of real-world walking activity are most important for cardiovascular risk in people with stroke.

A second line of work has focused on examining predictors of real-world walking activity in people with stroke [15, 28, 33–35]. This line of work has revealed that measures of walking capacity are strongly related to real-world walking activity after stroke. A recent meta-analysis by Thilarajah and colleagues found that the 6-Minute Walk Test, a measure of walking capacity, explains 37% of the variance in physical activity in people with stroke [15]. This suggests that measures of walking capacity are critically important and could potentially serve as a proxy for real-world walking activity in people with stroke. Real-world activity monitoring can be costly and cumbersome [36]; thus, if rehabilitation professionals were able to utilize laboratory-based measures of walking capacity as a proxy for real-world walking activity, this could potentially save time and resources. However, other work suggests that performance on laboratory-based measures of walking capacity does not necessarily translate to real-world walking behavior [18, 37–39]. Thus, whether real-world monitoring is more informative than laboratory-based measures of walking capacity in understanding the relationship with cardiovascular risk is not known at this time.

These lines of work have exposed two critical knowledge gaps. The first is that it remains unknown *which* measures of real-world walking activity are most important for cardiovascular risk in people with stroke. While previous studies have selected measures of *potential* importance, the first objective of this work was to determine *which* measures of real-world activity are most important to identify cardiovascular risk in people with stroke. Elevated systolic blood pressure (SBP) is an important cardiovascular risk factor and likely the strongest risk factor for stroke [3, 6, 40]. We therefore examined the relationship between measures of real-world walking activity and SBP. The second knowledge gap is that it is not known whether real-world monitoring is more informative than laboratory-based measures of capacity *in the context of cardiovascular risk*. Therefore, our second objective was to determine if measures of real-world walking activity would be associated with SBP after accounting for measures of walking capacity (6-Minute Walk Test, 6MWT). We hypothesized that average steps/day, average number of walking bouts/day, the percent time spent in sedentary behaviors, and the fragmentation index would be significantly associated with SBP and that these activity measures would be significantly associated with SBP after accounting for the 6MWT.

## Methods

### Study design and participants

This was a cross-sectional analysis of baseline data from a multisite clinical trial with four sites: University of Delaware, University of Pennsylvania, Christiana Care Health System, and Indiana University (NCT02835313) [41]. To be included in this analysis, the following eligibility criteria were employed: *Inclusion:* (1) Ages 21–85, (2) ≥ 6 months post stroke, (3) Able to walk at a self-selected gait speed of ≥ 0.3 m/s without assistance from another person (assistive devices allowed), (4) Resting heart rate between 40 and 100 beats/min, (5) Resting blood pressure between 90/60 and 170/90 mmHg; *Exclusion:* (1) Evidence of cerebellar stroke, (2) Other potentially disabling neurologic conditions in addition to stroke, (3) Lower limb Botulinum toxin injection < 4 months earlier, (4) Current participation in physical therapy, (5) Inability to walk outside the home prior to stroke, (6) Coronary artery bypass graft, stent placement, or myocardial infarction within past 3 months, (7) Musculoskeletal pain that limits activity, (8) Unable to provide informed consent as indicated by an inability to answer at least 1 orientation correctly (item 1b on the NIH Stroke Scale) and inability to follow at least one, two-step comment (item 1c on the NIH Stroke Scale). In addition, only participants with complete data for the activity measures, 6MWT and SBP were included in this analysis. All participants signed informed consent approved by the Human Subjects Review Board at the University of Delaware or their respective institution prior to study participation (protocol number 878153–50).

### Theoretical framework

**Fig. 1:**
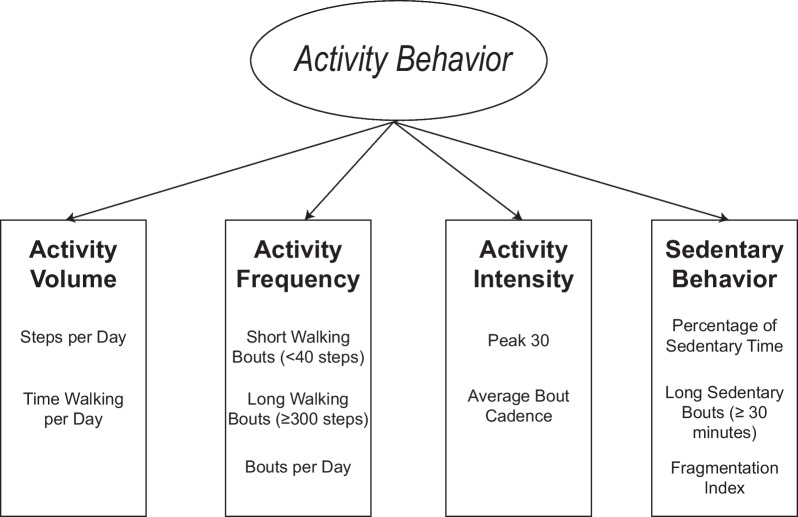
Theoretical Model. Activity behavior is comprised of four domains: Activity Volume, Activity Frequency, Activity Intensity, and Sedentary Behavior. The measures listed beneath each domain were considered measures of that domain in the current work

In order to determine which measures of real-world activity to include in our statistical analysis, we first conducted a review of the literature in people with stroke [2, 7–12, 14, 15, 17–19, 21, 22, 24, 25, 27, 28, 30, 34, 38, 42–47] and other populations [26, 32, 48–58] that measured real-world walking activity. This literature search resulted in over 30 different measures of real-world activity. We then systematically eliminated measures that were derivatives of each other (e.g., Peak 1, a measure of real-world activity intensity used in some studies [26, 52], is similar to Peak 30 [21, 52]) and measures that could be problematic in individuals with stroke. For example, METS is a common way that activity intensity is quantified [14, 27, 59, 60]; however, there are limitations to using METS in people with stroke [61–63]. Prior work has shown that individuals with stroke expend greater energy during walking compared to persons without stroke [63], suggesting that the use of METS in people with stroke may not be an accurate reflection of walking intensity. Once this smaller subset of measures was identified, the measures were then grouped under specific domains based on our knowledge of stroke and past literature suggesting that different activity measures assess different constructs [11, 12, 14, 22, 26, 32, 44]. Figure 1 provides a visual representation of the end result of this process and shows our theoretical framework for conceptualizing activity behavior. The model shows that activity behavior is comprised of four domains: *activity volume*, a*ctivity frequency*, *activity intensity*, and *sedentary behavior*. Each of these domains is intended to reflect an important but unique aspect of a person’s overall walking activity behavior. Table 1 displays the activity measures of interest, the domain of measurement, and how each measure was calculated.

**Table 1:** Activity measure calculations

Domain	Measure	Calculation
Activity volume	Average Steps/Day	$\frac{\#\mathrm{ steps taken over valid recording period}}{\mathrm{number of valid recording days}}$
	Average Time Walking/Day	$\frac{\#\mathrm{ minutes walking over valid recording period}}{\mathrm{number of valid recording days}}$
Activity frequency	Average Number of Short Bouts/Day (< 40 steps)	$\frac{\#\mathrm{ short walking bouts over valid recording period}}{\mathrm{number of valid recoridng days}}$
	Average Number of Long Bouts /Day (≥ 300 steps)	$\frac{\#\mathrm{ long walking bouts over valid recording period}}{\mathrm{number of valid recording days}}$
	Average Number of Bouts/Day	$\frac{\#\mathrm{ walking bouts over valid recording period}}{\mathrm{number of valid recording days}}$
Activity intensity	Peak 30	$\frac{\mathrm{average steps}/\mathrm{minfor }30\mathrm{ highest minutes each day}}{\mathrm{number of valid recording days}}$
	Average Bout Cadence	$\frac{\mathrm{sum of steps}/\mathrm{minfor all active bouts}}{\mathrm{number of active bouts}}$
Sedentary behavior	Percent Sedentary Time	($\frac{\#\mathrm{ sedentary minutes over valid recording period}}{\mathrm{total wear time over valid recording period}}$)*100
	Average Number of Long Sedentary Bouts/Day (≥ 30 min)	$\frac{\#\mathrm{ of long sedentary bouts over valid recording period}}{\mathrm{number of valid recording days}}$
	Fragmentation Index	$\frac{\mathrm{number of sedentary bouts }\ge 5\mathrm{minover valid recording period}}{\mathrm{total number of sedentary minutes over valid recording period}}$

*Activity volume* This domain is intended to capture a person’s overall volume of activity and encompasses measures such as averages steps/day (ASPD) [32] and time walking per day [24, 45] which provide an global representation of a person’s overall volume of activity over a particular period of time. Measures of activity volume, specifically ASPD, was associated with all-cause mortality in a large sample of adults living in the United States [52], suggesting that measures of activity volume may have important health implications. Furthermore, these measures can be readily extracted from a variety of step activity monitors which could facilitate their implementation in clinical settings. However, past work also suggests that measures of activity volume may be insufficient for understanding the relationship between activity and health and that additional or alternative measures are needed [12, 14, 24, 25, 30, 32].

*Activity frequency* Previous work suggests that the *frequency (i.e., bouts)* in which activity is accrued throughout the day differs in people with stroke compared to healthy controls [7, 24, 46]. In particular, past work has shown individuals with stroke engage in fewer overall bouts of walking activity [24, 46] and fewer long-distance bouts compared to older adults [24]. In addition, longitudinal studies in individuals with stroke have demonstrated that increases in activity volume (i.e., ASPD) may be partly explained by increases in the number of walking bouts [17, 64]. This suggests that the *frequency* in which activity is accrued may provide unique and important information beyond measures of activity volume (i.e., ASPD). The number of long (≥ 300 steps) [24, 46, 54] and short (< 40 steps) bouts [24, 46, 54] of walking activity as well as the overall number of walking bouts per day [24, 45, 46] were considered measures of activity frequency.

*Activity intensity* Stroke prevention guidelines suggest that individuals with stroke should engage in moderate-to-vigorous intensity aerobic physical activity to lower their risk of recurrent stroke and cardiovascular events [65]. This suggests that the cardiovascular *intensity* of walking activity may also be important when monitoring real-world walking behavior in people with stroke. In support of this point, Fini and colleagues found that greater time spent in moderate-to-vigorous physical activity was associated with a reduction in some cardiovascular risk factors in people with stroke over a two-year monitoring period [14]. This study, among others [22, 25, 43, 44], provide support that the cardiovascular *intensity* of real-world activity may be important in addition to the overall *volume* of activity. Measures of walking cadence have been utilized in prior studies as a proxy for the cardiovascular intensity of walking [21, 26, 32, 47, 52]. Thus, Peak 30 and average bout cadence (i.e., the average steps/minute across all bouts of walking) were considered measures of activity intensity in the current work [21, 32, 47, 52]. Peak 30 captures the 30 highest, but not necessarily consecutive, minutes of activity in a day and was previously shown to be associated with cardiometabolic risk factors in a large sample of adults [32]. Thus, Peak 30 was intended to capture an individual’s highest stepping activity in a day. Average bout cadence, on the other hand, was intended to capture a slightly different aspect of activity intensity by quantifying an individual’s average rate (i.e., cadence) of stepping during bouts of walking [47].

*Sedentary behavior* There is growing consensus that *sedentary time* is an independent construct of active time [7, 8, 10, 25, 27, 28, 50, 66]. Previous studies have shown that time spent in sedentary behaviors is associated with negative health outcomes, independent of active time [50, 67, 68]. Other studies have shown that breaking up the amount of time spent in sedentary behaviors has positive effects on cardiometabolic markers, such as blood glucose, systolic blood pressure and body mass index [30, 57, 58]. Taken together, these findings suggest that in addition to measuring time spent in active behaviors, time spent in sedentary behaviors should also be measured when attempting to understand the relationship between activity and cardiovascular risk. The percentage of time spent in sedentary behaviors [14, 28, 55, 56], the number of long (≥ 30 min) sedentary bouts [11, 14, 48], and the fragmentation index [25, 30, 57, 58] were considered measures of the sedentary domain. The fragmentation index is a measure that quantifies interruptions in sedentary behavior. Its calculation is shown in Table 1 where a higher value indicates more interrupted sedentary behavior. This measure was intentionally chosen based on prior literature demonstrating positive effects of breaks in sedentary time on cardiometabolic markers of health [30, 57, 58].

### Measures

During the baseline visit of the clinical trial, demographic information (i.e., age, gender, race) and stroke information (i.e., time since initial stroke) were collected. Participants’ resting blood pressure was collected in accordance with the American College of Sports Medicine (ACSM) guidelines [69]. Specifically, blood pressure readings were obtained with the participant seated in a chair with back support for at least 5 min, their legs uncrossed, and the arm supported at the level of the heart. A minimum of two readings were obtained with at least 1-min between readings. The two readings were averaged to represent the participant’s resting blood pressure [69]. However, if a difference of > 5 mmHg was observed between the first and second readings, an additional reading was obtained, and the average of these multiple readings was used.

To measure walking capacity, participants completed the 6-Minute Walk Test (6MWT). Participants were instructed to walk continuously as fast as possible for 6 min around a 42-m rectangular track [70]. Participants were instructed that they may stop and rest at any point during the test if needed but that the timer will continue. The 6MWT is a valid and reliable test of walking endurance in people with stroke [71, 72].

To measure real-world walking activity, participants were provided with a Fitbit One or Fitbit Zip to wear on their non-paretic ankle. The Fitbit has demonstrated acceptable accuracy in detecting stepping activity in people with stroke particularly when placed at the non-paretic ankle [73–76]. Participants were instructed to wear the device for 7 days; however, a minimum of 3 days of activity was required [20]. Participants were instructed to go about their usual activity while wearing the device and to remove it for water-based activities and sleep. Upon returning the device, a trained physical therapist inspected the data to ensure the minimum wear criteria was met. To determine valid recording days, the participant was queried about any inconsistencies or irregularities in the data. The days in which participants were issued and returned the device were not counted towards the 3-day minimum, nor were any days in which the participant did not wear the device during waking hours.

### Data processing

**Fig. 2:**
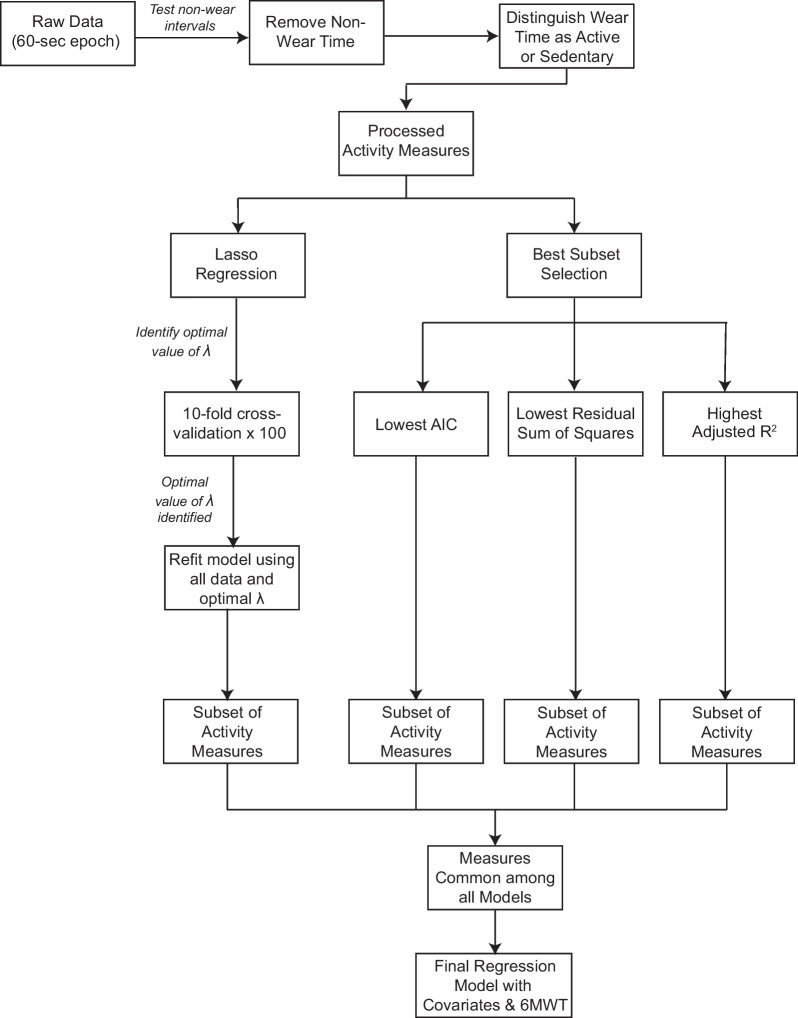
Data Pipeline. *6MWT* 6-Minute Walk Test

Figure 2 displays a data pipeline that demonstrates how the data were processed and analyzed. Participants’ step data was exported into 60-s sampling epochs to calculate the activity measures of interest [Fig. 2: “Raw Data (60-s epoch)”]. The first stage of data processing involved determining “wear” and “non-wear” time using the R package “accelerometry” [77]. Determining non-wear time is a critical decision when processing accelerometry data to reduce the risk of erroneously classifying sedentary time as non-wear time and vice-versa. We therefore employed a two-step process to determine an appropriate “non-wear” window and increase our confidence in this decision (Fig. 2: “Testing non-wear intervals”). First, non-wear windows of 3 h through 6 h were tested, and the number of sedentary and non-wear minutes were compared using a within-subjects analysis of variance (ANOVA) where the non-wear window was the within subjects variable. Post-hoc testing was conducted if the model was statistically significant. Second, a clinician with expertise in stroke rehabilitation independently coded whether each minute was “non-wear”, “sedentary” or “active” time for a random subset of 10 participants, and these results were compared to those of the different non-wear windows. These steps revealed significant differences (p < 0.05) in the number of sedentary and non-wear minutes for the 3-h non-wear window compared to all other non-wear windows. Comparing these results to the clinician responses revealed the highest agreement with the 4-h non-wear window (> 85% agreement for all 10 participants, mean agreement of 94.67%). We therefore determined the 4-h non-wear window was most appropriate. Under this definition, “non-wear” time was defined as any interval of at least 240 consecutive minutes (4 h) with 0 steps, allowing for 2 spurious minutes of activity of up to 2 steps each minute. Non-wear minutes were then removed from further analysis (Fig. 2: “Remove Non-Wear Time”). Any minutes that did not meet this criterion were defined as “wear” time. “Wear” time was further categorized as “active” or “sedentary” (Fig. 2: “Distinguish Wear Time as Active or Sedentary”). “Active” minutes were any minutes with at least 1 step, with the exception that a minute with only 1 step could not have a minute of 0 steps before and after it. All other “wear” minutes that did not meet this criterion were considered “sedentary” minutes. For example, a series of minutes with 0 steps, 1 step, 0 steps would be labeled: sedentary, sedentary, sedentary. A series of minutes with 10 steps, 12 steps, 0 steps, 20 steps would be labeled: active, active, sedentary, active. The activity measures were calculated from the “active” and “sedentary” time (Fig. 2: “Processed Activity Measures”). For example, the average time walking/day was calculated from the “active” minutes, and the percent sedentary time was calculated using “sedentary” minutes (Table 1).

### Statistical analysis

A two-step statistical analysis was employed to best address each of our study objectives. To address our first objective of identifying a subset of activity measures most strongly related to SBP, two variable selection techniques were utilized. For the primary analysis, lasso regression was employed. Lasso regression applies a penalty, controlled by the parameter λ, that shrinks the regression coefficients closer towards zero such that some of the variables (i.e., activity measures) are dropped from the model [78, 79]. The result is a simpler model containing a subset of variables whose coefficients were not zero. Those “surviving” variables are therefore interpreted as most strongly related to the outcome. For this work, the optimal value of λ was chosen using tenfold cross-validation which was replicated 100 times to achieve a stable solution (Fig. 2: “10-fold cross-validation × 100”) [78]. The optimal value of λ was considered the value associated with the smallest mean squared error on the test data [78, 79]. Once this optimal value of λ was identified, the model was then re-fit using all of the data and the optimal value of λ (Fig. 2: “Refit model using all data and optimal λ”). This process resulted in a subset of walking activity measures most strongly related to SBP. The lasso regression was performed using R Statistical Software (v3.6.1) [80] and the “glmnet” package [81].

To increase our confidence in the subset of activity measures retained from lasso, we also utilized the best subset method and compared these results to that of lasso. Unlike lasso, which performs variable selection by shrinking coefficients, the best subset method performs variable selection by fitting separate regression models for all possible combinations of predictors to determine which model (i.e., subset of predictors) is “best” [78, 82]. For this work, we determined which model was “best” by examining the AIC (Akaike information criterion), adjusted R^2^, and the model with the lowest residual sum of squares (Fig. 2) [78]. As lower AIC values indicate a better model, we rank-ordered all 1024 possible models from lowest to highest AIC and selected the model with the lowest AIC value [78]. As higher adjusted R^2^ values indicate better fit, we rank-ordered all possible models from highest to lowest adjusted R^2^ and selected the model with the highest adjusted R^2^ value [78]. As the residual sum of squares (RSS) always decreases as more variables are added to the model, we utilized the number of variables retained from lasso (*p*) and identified the best *p*-variable model with the lowest RSS. For example, if lasso retained 2 variables as most strongly related to SBP, we identified the best 2-variable model with the lowest RSS. The result of this step was three models (i.e., subsets of predictors) with the lowest AIC, highest adjusted R^2^, and lowest RSS. The best subset models were conducted using the regsubsets function within the “leaps” package [83] in R as well as the Regression Best Subsets extension in SPSS Version 28.0, Armonk, NY: IBM Corp. These results were compared to that of lasso (Fig. 2: “Measures Common among all Models”). Measures that were common among all approaches were fit in a separate linear regression model.

Sequential linear regression was used to address our second objective of understanding if the subset of activity measures selected were significantly related to SBP after accounting for walking capacity (Fig. 2: “Final Regression Model with Covariates & 6MWT”). In this approach, predictors are entered in blocks and the change in R^2^ value is evaluated after each block entry to determine if the block is significantly related to SBP after adjusting for the previous blocks [84]. The first block of predictors included covariates, specifically age, gender, race, and time since initial stroke. Gender was coded as male (0) or female (1). Race was categorized as white, black, and other which consisted of individuals who identified as races other than black or white (e.g., Asian) or identified as being more than one race. Race was then dummy coded as white compared to black and white compared to other. Walking capacity (i.e., 6MWT) was entered into the second block. The third block consisted of the common activity measures among lasso and best subset models. Thus, this two-step approach allowed us to discern the extent to which the activity measures were associated with SBP after accounting for covariates and walking capacity by evaluating the change in R^2^ value associated with each block of predictors which would not have been possible with a one-step approach. All regression assumptions were tested and met. The sequential linear regression was conducted in SPSS Version 28.0, Armonk, NY: IBM Corp.

## Results

Two-hundred and seventy-nine participants had complete data for the activity measures, 6MWT and SBP when this analysis was conducted. Three participants were removed due to systolic blood pressures below eligibility criteria, resulting in a final sample size of 276 participants. Table 2 displays the demographic and clinical characteristics of our study sample. The ASPD [9, 14, 21, 33] and percent sedentary time [10, 27, 85–87] of participants in our sample are comparable to samples in other reports.

**Table 2:** Demographic and clinical characteristics of study sample (n = 276)*

Characteristic	Participants
Age (years)	65.0 (IQR 17.0)
Gender (male/female)	Male: n = 143 (51.8%) Female: n = 133 (48.2%)
Time Since Initial Stroke (months)	23.0 (IQR 41.0)
Race	White: n = 169 (61.2%) Black: n = 64 (23.2%) Other: n = 38 (13.8%) Prefer Not to Respond: n = 5 (1.8%)
Systolic blood pressure (mmHg)	128.13 (SD 16.26)
Body mass index (kg/m^2^)	29.79 (IQR 8.19)
6-Minute Walk Test (m)	311.87 (IQR 181.85)
Number of valid step activity days	8.0 (IQR 5.0)
Average steps/day	4175.0 (IQR 3149.5)
Percent sedentary time	82.1 (IQR 11.04)

*Continuous variables that were normally distributed are presented as mean (standard deviation, SD) and non-normal variables are presented as median (interquartile range, IQR). *mmHg* millimeters of mercury, *kg/m*^*2*^ kilograms per squared meters, *m* meters

**Fig. 3:**
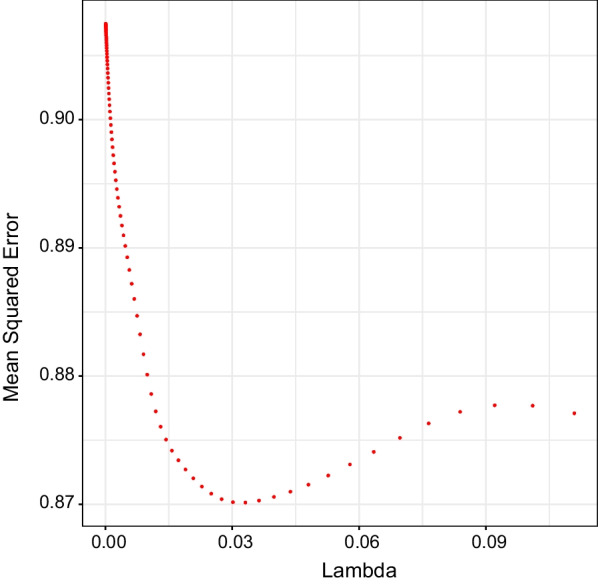
Relationship between Lambda and Mean Squared Error. The Y axis represents the mean squared error (MSE). The X axis represents values of lambda. The figure shows that as the strength of the penalty increases, MSE decreases to a point and then increases. The lambda value associated with the lowest MSE on the test data was 0.03. This point represents the lambda value at which model performance on the test data was best. The model was then refit using all data and this optimal value of lambda

**Fig. 4:**
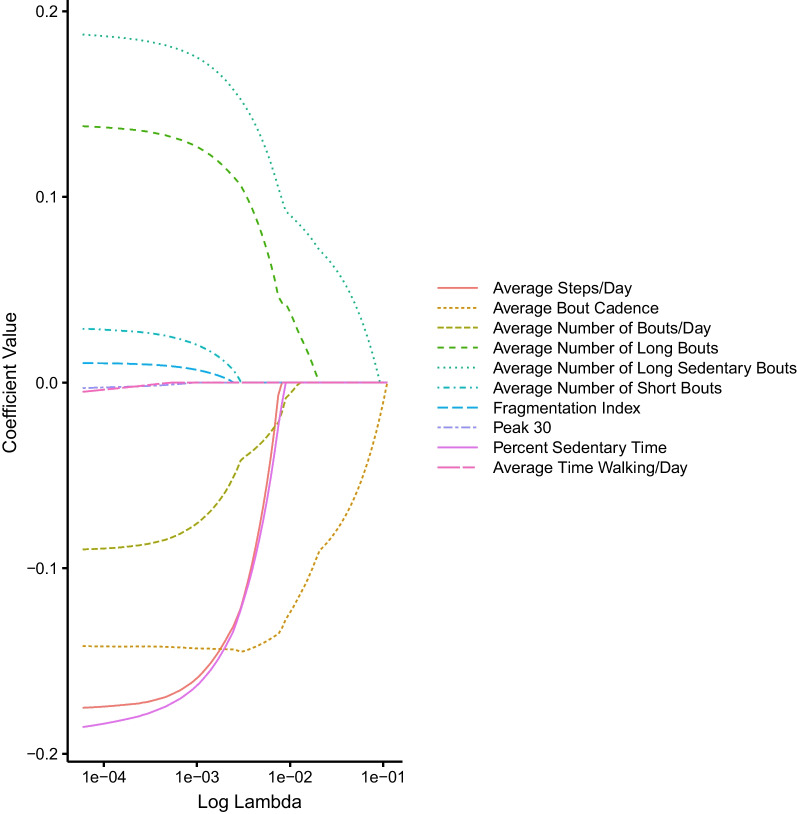
Coefficient Shrinkage with Increasing Lambda. The Y axis represents the value of the coefficients. The X axis represents values of lambda, where a higher value indicates a greater penalty (i.e., greater shrinkage). As lambda increases (i.e., from left to right on the X axis), the value of the coefficients shrink towards zero. The coefficient for Average Time Walking/Day is shrunk to zero first, followed by Peak 30, Fragmentation Index, Average Number of Short Bouts, Average Steps/Day, Percent Sedentary Time, Average Number of Bouts/Day, Average Number of Long Bouts, Average Number of Long Sedentary Bouts, and finally Average Bout Cadence. At a lambda value of 0.03, only Average Bout Cadence and Average Number of Long Sedentary Bouts remained in the model

Table 3 displays the subset of measures identified for the lasso and best subset models. For the lasso model, a λ value of 0.03 resulted in the lowest mean squared error (0.87) on the test data (Fig. 3). Figure 4 displays how the coefficients shrink with increasing λ values. Scanning the X-axis from left to right shows that the coefficient for average time walking/day was shrunk to 0 first, whereas the coefficients for long sedentary bouts and average bout cadence remained above 0 the longest (note: as λ approaches infinity, all coefficients are shrunk to 0). Refitting the model using all data and this value of λ resulted in only two activity measures whose coefficients were > 0, average bout cadence and long sedentary bouts. Thus, lasso regression selected average bout cadence and long sedentary bouts as most strongly related to SBP.

**Table 3:** Subset of activity measures identified using lasso regression and best subset models

Model	Model Performance	Subset of Activity Measures
Lasso	Optimal λ: 0.03 Mean Squared Error: 0.87	Average Bout Cadence, Long Sedentary Bouts
Best Subset: Lowest AIC	AIC: 1535.84	Average Bout Cadence, Long Sedentary Bouts
Best Subset: Lowest Residual Sum of Squares	Residual Sum of Squares: 70,495.52	Average Bout Cadence, Long Sedentary Bouts
Best Subset: Highest Adjusted R^2^	Adjusted R^2^: 0.02	Average Bout Cadence, Long Sedentary Bouts

The best subset models associated with the lowest AIC and highest adjusted R^2^ also contained only average bout cadence and long sedentary bouts (Table 3). As lasso identified two activity measures as most strongly related to SBP, we also examined the best two-variable model associated with the lowest RSS. This model also contained only average bout cadence and long sedentary bouts (Table 3). Thus, all models for best subset and lasso resulted in the same conclusion, that average bout cadence and long sedentary bouts were most strongly associated with SBP.

Average bout cadence and long sedentary bouts were then included in block 3 of the sequential linear regression model. Collectively, the block of covariates was significant (ΔR^2^ = 0.089, p < 0.001), suggesting the covariates explained a significant amount of variance in SBP (Table 4). The addition of walking capacity (i.e., 6MWT) did not significantly improve the model (ΔR^2^ = 0.002, p = 0.480). In support of our hypothesis, the activity measures explained a significant amount of variance in SBP after accounting for covariates and walking capacity (ΔR^2^ = 0.027, p = 0.020). The full model was significant (p < 0.001) and explained 11.8% of the variability in SBP. The coefficients for age (β = 0.219, p < 0.001), gender (β = − 0.121, p = 0.046), black race (β = 0.165, p = 0.008), and average bout cadence (β = − 0.159, p = 0.022) were significant (Table 5). These results suggest that higher systolic blood pressure was associated with older age, male gender, black race, and a slower average bout cadence.

**Table 4:** Linear regression model predicting systolic blood pressure

Variables	R^2^	Model p	ΔR^2^	ΔR^2^ p
Covariates (Age, gender, race, time since initial stroke)	0.089	< 0.001	0.089	< 0.001
Walking capacity (6-Minute Walk Test)	0.091	< 0.001	0.002	0.480
Activity measures (Average Bout Cadence, Long Sedentary Bouts)	0.118	< 0.001	0.027	0.020

**Table 5:** Standardized regression coefficients of linear regression model predicting systolic blood pressure

Variable	β	p
Age	0.219	< 0.001
Gender	− 0.121	0.046
Time Since Initial Stroke	− 0.057	0.338
Race: Black	0.165	0.008
Race: Other	− 0.045	0.454
6-Minute Walk Test	0.114	0.088
Average Bout Cadence	− 0.159	0.022
Long Sedentary Bouts	0.090	0.129

## Discussion

The two primary objectives of this work were to (1) determine a subset of real-world walking activity measures most strongly related to SBP in people with stroke and (2) determine if this subset of walking activity measures was significantly associated with SBP after accounting for measures of walking capacity (i.e., 6MWT). We found that average bout cadence (a measure of activity intensity) and the number of long sedentary bouts (a measure of sedentary behavior) were most strongly associated with SBP in people with stroke and that these measures were related to SBP after accounting for walking capacity. There are two primary take-home messages from this work: (1) in the context of cardiovascular risk average bout cadence and the number of long sedentary bouts are important measures of walking activity, and (2) laboratory-based measures of walking capacity are not sufficient for understanding the relationship between walking activity and cardiovascular risk in people with stroke.

While previous studies have selected measures that represent activity domains of interest, our objective was to determine *which* measures might be most important among a battery of measures that have been utilized in previous studies [2, 7–12, 14, 15, 17–19, 21, 22, 24–28, 30, 32, 34, 38, 42–58]. Our finding that a higher average bout cadence and less long sedentary bouts are most strongly associated with lower SBP suggests that these walking activity measures may be most important for cardiovascular risk in people with chronic stroke. Our finding that a higher average bout cadence, a measure of real-world walking intensity, was associated with lower SBP is supported by physical activity recommendations for people with stroke which suggest that the intensity of activity is important for maximal health benefits and cardiovascular risk reduction [2, 65]. This is also supported by a longitudinal study by Fini and colleagues that found the duration and bouts of moderate-to-vigorous physical activity [measured using metabolic equivalents (METS)] was associated with cardiovascular risk factors over a two-year monitoring period in people with stroke [14].

We also found that a greater number of long sedentary bouts was associated with a higher SBP. This is supported by past work in individuals with stroke [14] as well as past work in other populations demonstrating a dose–response relationship between the duration of bouts spent in sedentary behaviors and risk for cardiovascular disease [48]. This finding also supports the notion that sedentary time may be its own unique construct independent of active time [7, 8, 10, 25, 27, 28, 50, 66]. However, individually, long sedentary bouts was not significant in our regression model (Table 5). Therefore, our finding that the block representing the activity measures was significant in our model (Table 4) was likely being driven by average bout cadence, whose individual regression coefficient was significant. This is also supported by the lasso model in which average bout cadence was the last measure to be dropped from the model (Fig. 4). Collectively, this could suggest that if measurement options are limited, average bout cadence should be prioritized, followed by long sedentary bouts.

Despite its ubiquitous use in stroke rehabilitation clinical trials [15–19], ASPD was not found to be related to SBP in our sample of participants with stroke. One reason for this may be due to the fact that our outcome of interest for this work was SBP, a measure of cardiovascular risk. A common purpose for measuring ASPD in prior work has been to understand if a laboratory- or clinic-based intervention translates to changes in real-world walking behavior [16, 18, 37, 38], which was not the outcome of interest in the current work. This could suggest that how real-world walking activity is quantified may need to differ based on the context. For example, when attempting to understand how a laboratory or clinic-based intervention translates to real-world walking behavior or to obtain a global assessment of a stroke survivor’s walking activity levels, ASPD may be a reasonable choice. However, when attempting to understand how a stroke survivor’s real-world activity affects their cardiovascular risk, our results suggest that average bout cadence and long sedentary bouts may be superior measures. This suggests that careful consideration should be given to *which* measure(s) are used to quantify real-world walking activity depending on the purpose of measuring this behavior after stroke.

The activity measures (i.e., average bout cadence and long sedentary bouts) explained a significant amount of variability in SBP after accounting for covariates and 6MWT, which was not significantly associated with SBP. This result suggests that: (1) measures of real-world walking activity are distinct from laboratory-based measures of walking capacity, specifically the 6-Minute Walk Test, and that the two do not measure the same abilities post-stroke, and (2) an individual’s performance on the 6-Minute Walk Test is not sufficient for understanding the relationship between walking activity and cardiovascular risk in persons with stroke. Physical activity is related to important cardiovascular risk factors for stroke [1–5, 32], which is supported by the current work. Our results serve as an extension of prior work by demonstrating that this relationship cannot be inferred through laboratory-based assessments of walking capacity. Our real-world walking activity measures were calculated from numerous days of activity monitoring and are therefore likely a more accurate reflection of a person’s actual walking performance in the real world than a measure of walking capacity conducted at a single point in time in the laboratory. This is supported by our finding that the walking activity measures were significantly associated with SBP, whereas the 6MWT was not, and by previous work demonstrating that laboratory-based measures of walking capacity do not necessarily translate to real-world walking behavior [18, 37–39]. Taken together, these findings suggest that when attempting to understand (and possibly modify) real-world walking behavior as it relates to cardiovascular risk reduction, laboratory-based measures are likely insufficient and real-world monitoring is imperative.

### Limitations

There are several limitations to consider when interpreting the results of this work. First, we made several assumptions when determining non-wear time of the Fitbit device which could have affected our activity measure calculations. For example, a minute with 0 steps could be a minute of sedentary time or a minute of non-wear time. To increase our confidence in these assumptions, we tested multiple non-wear windows and leveraged the opinion of an expert clinician. Despite these efforts, it is possible that we incorrectly identified non-wear time as sedentary time and vice-versa in some cases. However, the median percent sedentary time in our sample (82.1%, IQR 11.04%) is comparable to previous reports in individuals with stroke [10, 27, 85–87], increasing our confidence in these assumptions. Second, we were constrained to a 60-s sampling epoch for our activity measure calculations which likely caused an underestimation of activity bouts [24, 88] and overestimation of time spent in active behaviors [88]. Future studies could consider replicating our procedures with a device that permits a smaller sampling epoch and compare results. Third, while cadence has been previously used to quantify the intensity of real-world activity in persons with stroke [10, 22, 29, 43] and other populations [26], this may not be the most appropriate measure of real-world activity intensity in people with stroke. Stroke often results in neuromotor impairments that can impact gait speed [89] and metabolic efficiency of gait [62, 63] which might confound the relationship between cadence and heart rate, which is why we did not hypothesize these measures to be most important. Thus, additional studies are needed to determine the relationship between cadence and heart rate in people with stroke. Fourth, this work is cross-sectional, and we therefore do not know if changes in average bout cadence and long sedentary bouts result in changes in SBP in people with stroke. Future longitudinal studies are needed to confirm our findings. Finally, we used a theoretical approach based on a review of the literature to develop our theoretical model (Fig. 1) and determine which activity measures to include in our statistical analysis. Alternatively, we could have utilized a data-driven approach to determine which activity measures to include in the analysis. Thus, future work may consider replicating our procedures using a data-driven approach and compare results. In a similar vein, future studies could consider empirically testing our theoretical model using confirmatory factor analysis to better understand the relationships between the activity measures and their hypothesized measurement domains.

## Conclusions

Measures of real-world walking activity, specifically average bout cadence and long sedentary bouts, were most strongly associated with SBP in people with chronic stroke. This suggests that the intensity of real-world walking and sedentary behavior may be important for cardiovascular risk in persons with stroke. These activity measures were associated with SBP after accounting for covariates and walking capacity, suggesting that real-world activity monitoring is critical for understanding the relationship between walking activity and cardiovascular risk in people with stroke and this relationship cannot be inferred from laboratory-based measures of walking capacity. Longitudinal studies are needed to determine if changes in average bout cadence and sedentary bouts affect SBP and cardiovascular risk in individuals with stroke.

## Data Availability

The data associated with this analysis are available from the corresponding author upon request.

## Abbreviations

ASPD: Average steps/day
SBP: Systolic blood pressure
6MWT: 6-Minute Walk Test
ANOVA: Analysis of variance
AIC: Akaike information criterion
RSS: Residual sum of squares
MSE: Mean squared error
METS: Metabolic equivalent of task
mmHg: Millimeters of mercury
kg/m^2^: Kilograms per squared meters
m: Meters
